# Correction to: Incidence and prognostic factors in severe drug-induced interstitial lung disease caused by antineoplastic drug therapy in the real world

**DOI:** 10.1007/s00432-022-04077-z

**Published:** 2022-06-02

**Authors:** Sawako Kaku, Hidehito Horinouchi, Hirokazu Watanabe, Kan Yonemori, Takuji Okusaka, Narikazu Boku, Naoya Yamazaki, Akira Kawai, Yuichiro Ohe, Masahiko Kusumoto

**Affiliations:** 1 Department of Diagnostic Radiology, National Cancer Center Hospital, 5-1-1 Tsukiji, Chuo-ku, Tokyo, 104-0045 Japan; 2 Department of Thoracic Oncology, National Cancer Center Hospital, 5-1-1 Tsukiji, Chuo-ku, Tokyo, 104-0045 Japan; 3 Department of Medical Oncology, National Cancer Center Hospital, 5-1-1 Tsukiji, Chuo-ku, Tokyo, 104-0045 Japan; 4 Department of Hepatobiliary and Pancreatic Oncology, National Cancer Center Hospital, 5-1-1 Tsukiji, Chuo-ku, Tokyo, 104-0045 Japan; 5 Department of Gastrointestinal Medical Oncology, National Cancer Center Hospital, 5-1-1 Tsukiji, Chuo-ku, Tokyo, 104-0045 Japan; 6 Department of Dermatologic Oncology, National Cancer Center Hospital, 5-1-1 Tsukiji, Chuo-ku, Tokyo, 104-0045 Japan; 7 Department of Musculoskeletal Oncology, National Cancer Center Hospital, 5-1-1 Tsukiji, Chuo-ku, Tokyo, 104-0045 Japan; 8 Course of Advanced Clinical Research of Cancer, Juntendo University Graduate School of Medicine, Tokyo, Japan

## Correction to: Journal of Cancer Research and Clinical Oncology 10.1007/s00432-022-03932-3

The first and 9th author’s affiliation details in the original article are incorrect.

The correct 1st author's affiliation is Department of Diagnostic Radiology, National Cancer Center Hospital, 5-1-1 Tsukiji, Chuo-ku, Tokyo 104-0045, Japan; Course of Advanced Clinical Research of Cancer, Juntendo University Graduate School of Medicine, Tokyo, Japan.

The correct 9th author's affiliation is Department of Thoracic Oncology, National Cancer Center Hospital, 5-1-1 Tsukiji, Chuo-ku, Tokyo 104-0045, Japan; Course of Advanced Clinical Research of Cancer, Juntendo University Graduate School of Medicine, Tokyo, Japan.

The "lower gastrointestinal cancer" is incorrectly written as "upper gastrointestinal cancer" in the original article published. The correct sentence is, "The following most common sites of primary cancer were hepatobiliary and pancreatic cancer (9.2%), breast cancer and upper gastrointestinal cancer (8.3%), lower gastrointestinal cancer, and melanoma (6.7%)".

The Original Article is updated.

